# Seymour fracture management and functional outcome assessment: a case report

**DOI:** 10.1093/jscr/rjaf083

**Published:** 2025-02-27

**Authors:** Victor Hugo Garzón Ortega, Alfonso Iván Sánchez Terán

**Affiliations:** Facultad de Medicina, Universidad Nacional Autonoma de Mexico, Ciudad Universitaria, Mexico City, Mexico; Department of Plastic and Reconstructive Surgery, Hospital General “Dr. Manuel Gea González”, Calzada de Tlalpan 4800, Colonia Belisario Domínguez, Sección XVI, Delegación Tlalpan, C.P. 14080, Mexico City 14000, Mexico; Facultad de Medicina, Universidad Nacional Autonoma de Mexico, Ciudad Universitaria, Mexico City, Mexico; Department of Plastic and Reconstructive Surgery, Hospital General “Dr. Manuel Gea González”, Calzada de Tlalpan 4800, Colonia Belisario Domínguez, Sección XVI, Delegación Tlalpan, C.P. 14080, Mexico City 14000, Mexico

**Keywords:** distal phalanx, infection, juxta-epiphyseal fracture, osteomyelitis, phalanx fracture, Seymour fracture, case report

## Abstract

Seymour fracture, a rare entity whose management is complex due to the mechanism and rate of complications. We present a case to describe our management approach. A 14-year-old child with a laceration secondary to a boot crush of 1 day of evolution. The physical examination reveals a lacerated fifth digit involving skin, nail bed, and distal phalange. Radiography was performed, Salter-Harris I fracture was diagnosed. Early surgical intervention was performed, irrigation, nail bed repair, debridement, open reduction, and osteosynthesis with K-wires. The patient was referred to rehabilitation and an antibiotic scheme of amoxicillin-clavulanic plus clindamycin was given. After 8 weeks Kirschner wire was removed, and no complications were reported. A fingertip injury outcome score was performed obtaining 11 points. Early intervention is a must. The use of Fingertip Injuries Outcome Assessment Score is vital for followup in children. Collaboration across disciplines is key to improving outcomes.

## Introduction

Seymour fractures are a type of open juxta-epiphyseal fracture of the distal phalanx, primarily affecting children [1]. The management is critical due to the high risk of complications, including osteomyelitis, if not treated promptly [2, 3].

The presentation is associated with flexion deformity and ungual subluxation mimicking a mallet finger. Due to the definition established in 1966, identifying becomes challenging in the presence of open fractures.

Pediatric fractures should take into account, the open growth plate, and therefore requiring special consideration regarding immobilization and remodeling potential [4, 5]. This can be managed with conservative treatment, nonetheless, we should take into account the mechanism of injury, probability of infection, and the possibility of hand functionality impact in medical management only [2].

### Patient information, clinical findings, and timeline

**Figure 1:**
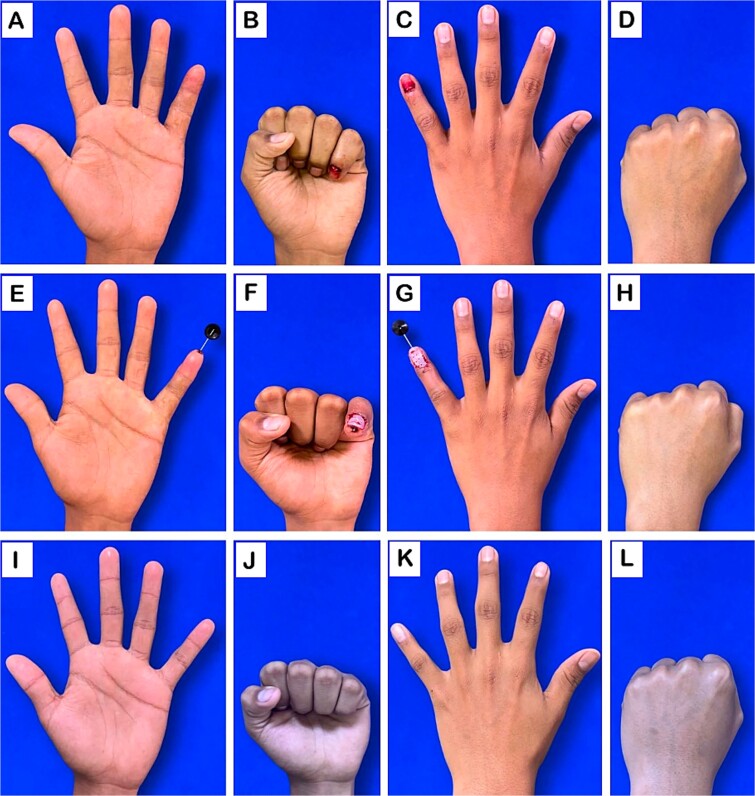
Clinical evaluation of the hand. (A–D) Preoperative images: volar and dorsal views. (E–H) Postoperative images following surgical intervention: volar and dorsal views. (I–L) images at 10-month follow-up: volar and dorsal views.

**Figure 2:**
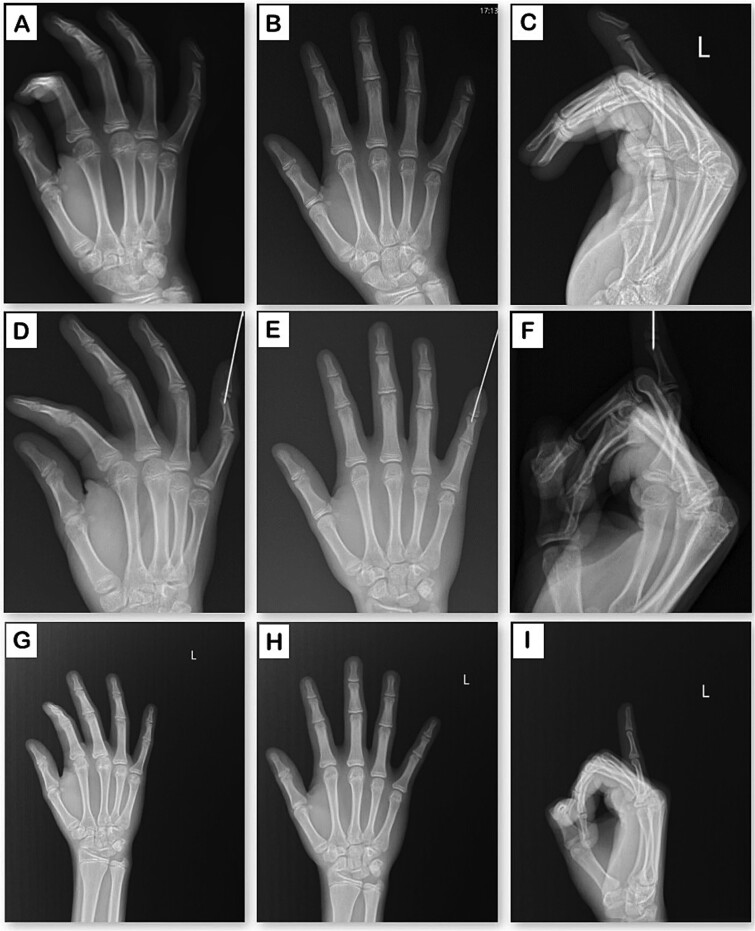
Radiographic evaluation of the hand in three planes. (A–C) Preoperative X-rays: oblique, anteroposterior, and lateral views. (D–F) Postoperative X-rays following surgical intervention: oblique, anteroposterior, and lateral views. (G–I) X-rays at 10-month follow-up: oblique, anteroposterior, and lateral views.

A previously healthy 14-year-old child comes to our service and during the physical exam: A laceration with blunt edges on the fingertip of the left fifth finger, involving the skin, nail plate, nail bed, and bone (Fig. 1). X-ray was performed in oblique, lateral, and anteroposterior views, showing a displaced Salter-Harris type I fracture (Fig. 2).

The patient sustained blunt-force trauma on the left hand on March 16 and presented to our service on March 17. Radiography confirmed the injury, and surgical intervention was performed, including irrigation, nail bed repair, debridement, open reduction, and K-wire osteosynthesis. The patient was discharged with antibiotics and referred to rehabilitation. A follow-up on March 25 showed normal healing, and the K-wire was removed on May 15 without complications.

### Intervention

Due to the late presentation, we prescribe amoxicillin-clavulanic acid plus clindamycin. The treatment followed a meticulous surgical approach, addressing both the fracture and associated soft tissue injuries under local anesthesia to ensure optimal conditions for repair. A thorough exploration of the wound was undertaken to evaluate the extent of soft tissue damage. The rest of the nail plate was removed to gain better access, facilitating proper irrigation and selective debridement of devitalized tissue to minimize infection risk and promote healing. The fracture was then reduced restoring the anatomical alignment.

Stabilization was achieved through the insertion of K-wires across the fracture site in a straight position, maintaining alignment and balance of the different insertion sites of flexor and extensor tendons. We did not consider using a technique where the DIP joint is fixed in a flexed position with X-wire fixation as it is more associated with vascular injury and bone avulsion. The nail bed, critical for proper postoperative nail growth, was meticulously repaired to prevent nail dystrophy or deformities. The surgical wound was subsequently closed in layers and a sterile dressing was applied (Fig. 1).

Postoperatively, the patient was monitored closely for signs of infection or compromised circulation. Wound care instructions were provided, and a follow-up appointment was scheduled for K-wire removal and assessment. This intervention was designed not only to address the immediate injury but also to minimize the risk of long-term complications.

We referred our patient to our rehabilitation department. On 8 weeks follow-up, the patient had increased strength and range of motion and a Fingertip Injury Outcome Score (FIOS) of 11. This score was determined as follows: nail normal (1), finger length (1), well-padded pulp (1), united bone (1), satisfactory cosmesis (1), 7–10 mm sensation (2), no pain (1), 75%–100% range of motion (1), 90% grip strength (1), and return to school (1).

## Discussion

The management remains a topic of debate, as there is no universally accepted protocol. Some centers advocate for conservative methods and finger casting. This variability extends to antibiotic administration, with physicians choosing different regimens based on individual clinical judgment.

We proposed a treatment protocol that included early intervention through washout, debridement, and open stabilization using K-wires. We opted for amoxicillin-clavulanic acid, with the addition of clindamycin in cases of delayed presentation. This choice aligns with the literature suggesting that appropriate antibiotic coverage is crucial in minimizing complications such as osteomyelitis [6–8].

Despite the early intervention, digital tip injuries can still lead to various complications. Delayed healing is a common issue that may arise from inadequate initial debridement or excessive tension, also leading to atrophic changes and hypersensitivity in the affected fingers. Additionally, deformities such as ‘hook nail’ can occur due to a shortened nail bed, leading to pain and discomfort that impact daily activities [7].

Other potential complications include flap loss and the risk of osteomyelitis which poses a serious threat [7]. These outcomes underscore the necessity for meticulous management and diligent follow-up. Although most patients achieve good functional outcomes, rehabilitation is the most critical part, and the greatest functional recovery will depend on it. Considering the patient’s age of 14, we used the short arc active range of motion protocol leading to full recovery of the ROM.

Assessing long-term outcomes relies on both qualitative and quantitative measures, such as those outlined in the FIOS. This score evaluates postoperative finger length, bone consolidation, nail aesthetics, sensation, range of motion, grip strength, and return to school. However, it is important to note that the validity of FIOS has not yet been established in the Mexican population. Future studies should aim to assess the reliability and applicability of this outcome measure [9]. The use of scoring systems can facilitate comprehensive assessments of functional recovery and ultimately improve patient.

## Conclusion

Our case highlights the importance of early intervention in reducing complications. Multidisciplinary management enhances functional recovery in affected patients. Moreover, further research is essential to investigate the long-term outcomes of these injuries. Utilizing the FIOS not only enhances patient care but also optimizes treatment strategies by providing measurable outcomes.
